# Testing and Optimizing Guided Thinking Tasks to Promote Physical Activity: Protocol for a Randomized Factorial Trial

**DOI:** 10.2196/40908

**Published:** 2022-09-08

**Authors:** Austin S Baldwin, Colin L Lamb, Bree A Geary, Alexis D Mitchell, Chrystyna D Kouros, Sara Levens, Laura E Martin

**Affiliations:** 1 Department of Psychology Southern Methodist University Dallas, TX United States; 2 Department of Psychological Science University of North Carolina at Charlotte Charlotte, NC United States; 3 Department of Population Health University of Kansas Medical Center Kansas City, KS United States

**Keywords:** physical activity, optimization, brief intervention, episodic future thinking, positive affective imagery, planning, exercise

## Abstract

**Background:**

Insufficient physical activity is associated with various health risks; however, most current physical activity interventions have critical barriers to scalability. Delivering interventions via technology and identifying active and inert components in early-phase development are ways to build more efficient and scalable interventions. We developed a novel intervention to promote physical activity that targets 3 brief guided thinking tasks, separately and in combination, using brief audio recordings: (1) episodic future thinking (EFT), (2) positive affective imagery (PAI), and (3) planning.

**Objective:**

The aim of this GeT (Guided Thinking) Active study is to optimize a scalable guided thinking intervention to promote physical activity using principles of the Multiphase Optimization Strategy (MOST). Mechanism-focused analyses will inform which components are optimal candidates for inclusion in an intervention package and which need refinement.

**Methods:**

We will enroll 192 participants randomized to receive intervention components delivered via an audio recording that they will listen to prior to weekly in-lab physical activity sessions. Participants in the high dose conditions will also be instructed to listen to the audio recording 4 additional days each week. We will evaluate effects of the components on physical activity over 6 weeks in a 2 (EFT vs recent thinking) × 2 (PAI vs neutral imagery) × 2 (planning vs no planning) × 2 (dose: 5×/week vs 1×/week) full factorial randomized trial.

**Results:**

The National Cancer Institute funded this study (R21CA260360) on May 13, 2021. Participant recruitment began in February 2022. Data analysis will begin after the completion of data collection.

**Conclusions:**

The GeT Active study will result in a scalable, audio-recorded intervention that will accelerate progress toward the full development of guided thinking interventions to promote physical activity.

**Trial Registration:**

ClinicalTrials.gov NCT05235360; https://clinicaltrials.gov/ct2/show/NCT05235360

**International Registered Report Identifier (IRRID):**

DERR1-10.2196/40908

## Introduction

### Background

Insufficient levels of regular physical activity, defined as less than 150 minutes a week of moderate-to-vigorous physical activity (MVPA), are associated with numerous health risks, including cardiovascular disease, obesity, and various cancers [1-7]. Nearly 50% of adults in the United States report insufficient levels of physical activity, and 26.6% report no regular activity [8]. Objective assessments indicate that rates of physical activity are even lower than self-reported rates [9,10]. Most current physical activity interventions require significant resources that create barriers to scalability (eg, staff time, significant participant burden) [11-15]. Most interventions are also “black boxes,” in that they include multiple intervention components (eg, *M*=8.4 components among 26 interventions [16]) without knowing which specific components are active and which are inert. This also creates barriers to efficiently disseminating the intervention and refining ineffective components [15]. Therefore, there is a need for novel intervention strategies to promote regular physical activity that are both effective and scalable.

Delivering physical activity interventions via technology-based platforms can address scalability and dissemination barriers by minimizing cost and resource demands. Physical activity interventions delivered via smartphone apps, websites, and audio recordings [12,13,17] have proven to be feasible and effective [12-14,18,19]. However, effective technology-based interventions [14,18] have the same “black box” problem as other interventions. Identifying active and inert intervention components in early-phase development (versus later on in the process) is a means to build more efficient and scalable interventions and is aligned with current intervention development frameworks, such as the Multiphase Optimization Strategy (MOST) [15], Obesity-Related Behavioral Intervention Trials (ORBIT) model [20], and Science of Behavior Change [21].

We developed a novel intervention to promote physical activity that targets 3 brief intervention techniques, separately and in combination. Episodic future thinking (EFT) is a guided time-perspective task that directs individuals to actively imagine themselves in the future at a meaningful event [22-27]. Positive affective imagery (PAI) is a guided imagery task to increase positive affective associations with a target behavior and reframe related physical and physiological experiences as positive [22,26,28]. Planning is a guided task in which individuals specify when, where, and how a target behavior will be enacted [29-31].

In developing and optimizing a scalable physical activity intervention, EFT, PAI, and planning have several advantages as potential intervention components. First, each component can be delivered as a brief intervention (ie, <3-4 minutes each). Lack of time is a common barrier to physical activity [32,33]; therefore, brief intervention components are optimal. Given their brevity, these components can be used in a delivery mode (ie, guided audio recording) that is easily scaled and disseminated. Second, EFT, PAI, and planning have demonstrated positive effects on physical activity and other health behaviors [17,34-41]. EFT is a novel intervention technique for physical activity but has been shown to influence dietary behavior [22,26,28] and smoking reduction [42].

**Figure 1:**
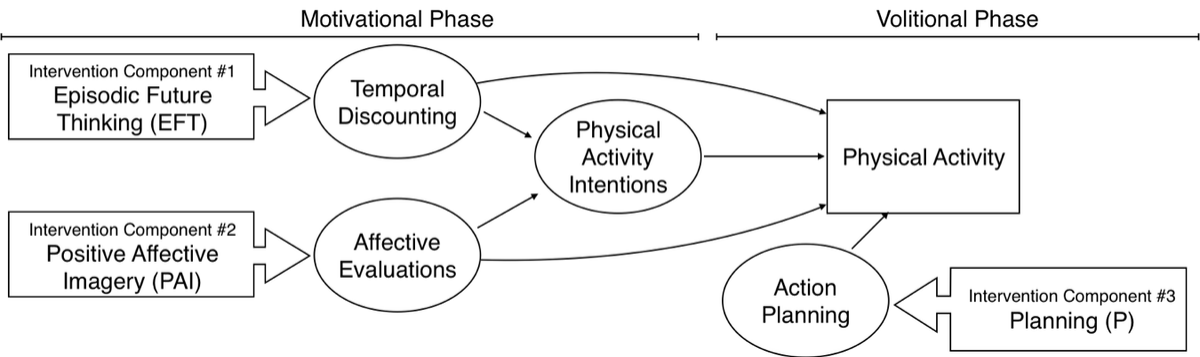
Conceptual model of intervention components, putative mechanisms, and behavior change phases.

Third, each component targets a different putative mechanism and thus different barriers to activity (Figure 1). A key motivational barrier to activity is the temporal trade-off that exists between health benefits of physical activity that are temporally distal (eg, weight control, disease prevention) and costs that are immediate (eg, time, physical exertion [43-45]). EFT targets and reduces temporal discounting rates and preference for immediate versus delayed reward in smoking and dietary choices [23,42]. Experiencing physical activity as affectively unpleasant is a common barrier to regular activity [46-48]. PAI can positively influence affective evaluations of physical activity [49]. Action planning is hypothesized to result from the planning intervention component. Action planning reliably predicts physical activity [30,50-52] and addresses a critical barrier to physical activity change: intentions not reliably leading to activity [53-55]. Finally, the components target mechanisms in 2 distinct phases of physical activity change: (1) motivational and (2) volitional (Figure 1) [56,57]. Targeting components in both phases is expected to lead to greater activity because both motivational and volitional processes are needed to change behavior [56]. Therefore, we expect a combination of components to have a stronger effect than any 1 component alone.

### Optimization of the Novel Intervention

To optimize the intervention with EFT, PAI, and planning, the unique and combined effects of the components need to be compared as they have previously only been examined separately, and their combined effects on physical activity are unknown. Three possible effects have implications when deciding which combination of components is optimal [15]: (1) independent effects, in which one component’s effect does not depend on another component; (2) synergistic effects, in which a component’s effect is strengthened by the presence of another component; and (3) antagonistic effects, in which a component effect is weakened by the presence of another component.

Comparing different frequencies of intervention use (ie, dosage) and how different doses influence the effects of EFT, PAI, and planning provides additional information for determining optimal combinations (eg, whether the effects of some components are strengthened by more frequent use). Optimal dosage of physical activity interventions is typically unexamined and a part of the “black box.” Determining mechanisms of the intervention components also accelerates the optimization process. We will use an experimental medicine approach [21,58] to test the extent to which (A) each component changes its putative mechanism, and (B) each mechanism is prospectively associated with physical activity, with the aim of elucidating why the intervention components are (or are not) effective [59].

### Objective of the GeT (Guided Thinking) Active Study

The overall objective of the GeT Active study is to optimize a scalable guided thinking intervention to promote physical activity, using principles of the MOST framework [15]. We will evaluate effects of the components on physical activity over 6 weeks in a 2 (EFT vs recent thinking) × 2 (PAI vs neutral imagery) × 2 (planning vs no planning) × 2 (dose: high vs low) full factorial randomized trial. We will use the following criteria to evaluate findings and identify the optimal combination of intervention components: effect of components on physical activity, efficiency (ie, identify inert components, antagonistic effects), participant burden (ie, total recording length, intervention dose), and acceptability of each guided thinking component. Mechanism-focused analyses will inform which components are optimal candidates for inclusion and which need refinement or reconsideration [59].

## Methods

### Intervention Component Evaluation

We plan to enroll 192 participants who will be randomized to receive the intervention components delivered via an audio recording that they will listen to prior to the weekly in-lab physical activity sessions. Participants in the high dose conditions will also be instructed to listen to the audio recording 4 additional days during each week. We will use a full factorial design whereby participants will be randomized to 1 of 16 combinations of the 4 intervention components (Table 1). The 4 intervention components are listed as follows.

The first intervention component is episodic future thinking (EFT). In the baseline session, participants will complete an interview with a research assistant (RA) to identify and imagine a positive event that is important to them and that they will experience 4-6 months into the future. In the audio recording (2 minutes and 35 seconds in length), participants will be prompted to imagine themselves at that future event in specific and vivid detail (eg, where they will be, what they are doing, how they are feeling), including imagining they have accomplished their goal of becoming more physically active over the preceding months. The content is similar to EFT tasks that have positively influenced food choice [22,28]. Participants not assigned to receive the EFT audio recording will be guided in an episodic recent thinking (ERT) interview and audio-recorded task (2 minutes and 45 seconds in length) to think in similar detail about a regular habit they enjoy and engaged in during the past week.

The second intervention component is positive affective imagery (PAI). Participants will be guided by the audio recording (3 minutes and 50 seconds in length) to think about positive feelings and associations with physical activity in specific, personal, and positive detail. They will imagine themselves engaging in physical activity, reframing physical sensations with positive attributions, and feeling satisfied about the challenge and accomplishment of physical activity. The content is similar to PAI tasks in previous studies [17,28,34]. Participants not assigned to PAI will receive a neutral imagery recording (2 minutes and 20 seconds in length) and will be guided to imagine specific physical sensations (ie, movements, muscles used) of a routine, daily (nonphysical) activity.

The third intervention component is planning. Participants will be guided by the audio recording (2 minutes and 35 seconds in length) to think about the remainder of their week and when (ie, days, times), where (ie, walking path, gym), and how (ie, walking with friend, alone) they plan to attain their remaining MVPA to reach the weekly 150-minute goal. The content is similar to planning tasks that have been shown to influence physical activity [38,40,41]. Participants not assigned to receive the planning component will not receive a control recording (ie, planning vs no planning).

The fourth component is the intervention dose. Participants will receive their assigned combination of intervention components either once a week during the in-person visit (low dose) or 5 times a week (in-person visit plus 4 additional days; high dose). Participants in the high dose conditions will receive instructions on how to access their audio recording file and will be assigned 4 additional days, including 1 weekend day, to listen to their audio recording. They will receive text message reminders to listen to the recording on those 4 days.

**Table 1:** Experimental condition assignments in the full factorial design.

	Experimental condition assignments															
Component	1	2	3	4	5	6	7	8	9	10	11	12	13	14	15	16
EFT^a^	Y^b^	Y	Y	Y	N^c^	N	N	N	Y	Y	Y	Y	N	N	N	N
PAI^d^	Y	Y	Y	Y	Y	Y	Y	Y	N	N	N	N	N	N	N	N
Planning	Y	Y	N	N	Y	Y	N	N	Y	Y	N	N	Y	Y	N	N
Dose	Hi^e^	Lo^f^	Hi	Lo	Hi	Lo	Hi	Lo	Hi	Lo	Hi	Lo	Hi	Lo	Hi	Lo

^a^EFT: episodic future thinking.

^b^Y: yes.

^c^N: no.

^d^PAI: positive affective imagery.

^e^Hi: high.

^f^Lo: low.

In each audio recording, the guided thinking components always appear in the following order: EFT/ERT, PAI/neutral imagery, and planning. We selected this order to minimize the cognitive load that temporal switching between tasks will require. This order allows listeners to imagine a future (or past) event first and then switch to the guided thinking tasks that are more present-focused. Planning is the final guided thinking component because it is designed to leverage the increased motivation for physical activity targeted by the other components and focus on enacting physical activity (ie, volitional phase). Table 2 includes examples of key pieces from each of the audio recording scripts.

**Table 2:** Examples of key pieces from each of the audio recording scripts.

Audio recording	Examples of key script pieces
EFT^a^	“Think back to the specific, positive event that you identified during your initial visit. You are looking forward to this event that will happen several months from now.” “Now, imagine that you are at this event, and you have accomplished your goal of becoming more physically active.” “Imagine the details of the event as specifically and vividly as you can…”
ERT^b^	“Think about the routine activity that you identified during your initial visit. This is a routine activity or habit that you do every week.” “Imagine the details as specifically and vividly as you can, as if it were happening again right now…”
PAI^c^	“Select an aerobic physical activity, such as brisk walking or hiking. Imagine yourself doing this activity today.” “Think about the positive benefits of this activity for you…how it might enable you to do more than you could before, and make your daily life feel more enjoyable…” “Imagine yourself doing this activity as vividly as you can…your body might feel warm…you might feel fatigued…the increase in sensations can be a good thing…your body is responding to the challenge you are giving it and becoming stronger, healthier, energized…” “Imagine that you’ve finished your activity today and you feel satisfied, confident, and energized…”
Neutral imagery	“Imagine you are doing a simple task or activity, an activity that you may do every day and that does not require much effort, such as folding laundry, household shopping, getting dressed for the day, or making your bed…” “Imagine yourself completing this activity from start to finish as vividly as you can…imagine your body moving and using your body to complete the task.” “What muscles are you using to complete this task? Do you feel your muscles contracting?”
Planning	“Think about where you are currently in your week and how many minutes of activity you still need to attain your goal of two and a half hours…” “What days of the week can you most easily schedule physical activity? What time during these days can you realistically engage in activity?...Where will you do the activity?...How will you do it? Will you be alone or with a friend?”

^a^EFT: episodic future thinking.

^b^ERT: episodic recent thinking.

^c^PAI: positive affective imagery.

### Study Design

We will use a 2 (EFT vs recent thinking) × 2 (PAI vs neutral imagery) × 2 (planning vs no planning) × 2 (dose: high vs low) full factorial design with a 1:1 allocation ratio to evaluate the components and their combinations in this randomized trial. This is not a 16-arm trial requiring comparison on individual experimental conditions; instead, the design allows for comparisons of means computed across aggregates of experimental conditions (ie, each comparison will involve all 16 experimental conditions). For example, the main effect of the EFT component will be tested by comparing the mean MVPA for the half of the sample who receive the EFT component (ie, those in conditions 1, 2, 3, 4, 9, 10, 11, and 12 in Table 1) to the mean MVPA for the half of the sample who do not receive EFT (ie, those in conditions 5, 6, 7, 8, 13, 14, 15, and 16 in Table 1).

### Ethics Approval

The study was approved by the Southern Methodist University Institutional Review Board (H21-003-BALA) on January 14, 2021.

### Eligibility

The inclusion criteria are that participants must (1) be 18-64 years of age, (2) be capable of providing informed consent, (3) have access to a smartphone, (4) be willing to attend all study visits and comply with the protocol, (5) be conversant in English, and (6) not currently meet recommended physical activity guidelines (defined as <150 minutes/week of self-reported MVPA). For safety considerations that could make moderate-intensity activity unsafe [60], we will exclude participants who report any of the following conditions: coronary artery disease, stroke, chronic obstructive pulmonary disease, chronic bronchitis, emphysema, diabetes, BMI>40, or orthopedic problems that limit physical activity.

### Recruitment

We will recruit 192 community-dwelling adults who are not currently meeting recommended physical activity guidelines. Recruitment strategies will focus on online postings and social media advertisements on various platforms (eg, Facebook, Instagram, NextDoor, Craigslist) in the Dallas-Fort Worth area. We will also actively recruit adults from traditionally underrepresented groups through advertising and postings in online outlets and community facilities that serve racial and ethnic minority groups.

### Screening

Initial eligibility will be determined with an online prescreen questionnaire, in which potential participants will report their physical activity for a typical week over the past 6 months using items from the International Physical Activity Questionnaire (IPAQ) [61]. They will also report on the inclusion criteria and exclusionary health conditions. Eligible participants will be contacted to schedule a telephone screen and a baseline study visit and to complete an initial COVID-19 screen in which they will be asked to self-report COVID-19 symptoms and their vaccination status.

On the day prior to the baseline study visit, we will assess participants’ physical activity during the past week using the telephone-based 7-day Physical Activity Recall (PAR), a valid and reliable measure of physical activity [62]. Participants who report >150 minutes of MVPA on this assessment will be excluded from participation. We will conduct this assessment on the day prior to the baseline visit to avoid turning away ineligible participants after they have already shown up in person. This assessment will serve as the baseline measure of participants’ physical activity.

### Randomization

We will stratify randomization based on 2 levels of baseline MVPA to explore the extent to which baseline MVPA moderates intervention effects: (1) individuals who report <60 minutes of weekly MVPA (ie, inactive or underactive) and (2) those who report between 60 and 149 minutes of weekly MVPA (ie, insufficiently active). These MVPA cutoffs reflect meaningful distinctions in current physical activity recommendations [1]. We will aim to recruit and enroll an equal number of individuals from both groups. Within each MVPA group, we will use block randomization with block sizes of 16 (ie, the total number of experimental conditions). We will use a random number generator to determine the random sequence within each block. The principal investigator will generate the random allocation sequence for each stratified randomization grouping. An RA will enroll and assign participants to their study condition.

### In-person Visits

There will be a total of 7 in-person visits over the 6-week study period. During these visits, participants will listen to their assigned audio recording, engage in a supervised 30-minute moderate-intensity walk on a treadmill, and complete study measures.

#### Baseline Visit

After completing the informed consent process, all participants will receive the same physical activity prescription (ie, >150 MVPA minutes/week) that is consistent with current recommendations [1]. A trained RA will provide instructions about increasing regular physical activity (eg, weekly recommendations, modes of activity) to attain 150 MVPA minutes/week. Participants will be instructed to focus on brisk walking to reduce potential variability in responses to different modes of physical activity and because walking is the preferred mode of activity among the general population [63]. All participants will be instructed to exercise at a moderate intensity.

Prior to listening to their assigned audio recording, participants will complete a baseline measure of study variables and demographics. Participants will then listen to their assigned audio recording before completing the in-lab brisk walking session. The audio file will be stored on a secured website and accessed via a tablet. Participants will be instructed to sit comfortably, close their eyes, and pay close attention to the guided thinking tasks. After listening to the audio recording and before walking, participants will complete assessments of the putative mechanisms.

Participants will receive instructions on how to wear a hip-worn accelerometer (ActiGraph wGT3X-BT) and then complete a supervised 30-minute brisk walk on a treadmill. Participants will also wear a heart rate monitor to ensure their walking intensity remains in the moderate range (ie, 64%-76% of their estimated maximal heart rate calculated using the formula: 220 − age). We will instruct participants to use the walking intensity to guide their unsupervised walking sessions during the remainder of the week. Participants will complete assessments of affective response, perceived exertion, and arousal during and immediately postexercise. Following the brisk walking session, participants will be reminded to aim for an additional 120 minutes (2 hours) of activity during the upcoming week, wear the accelerometer every day, listen to the audio recording on the designated days (high dose conditions).

#### Weekly Visits

During the weekly visit, the previous week’s data from the accelerometer will be downloaded and recorded. The RA will then conduct a 7-day PAR with participants to assess self-reported physical activity over the previous week. Participants in the high dose conditions will be asked to report their adherence to listening to the audio recording. The remainder of the weekly visits will follow the same procedure as the baseline visit. In the visits at weeks 3 and 6, participants will also complete a questionnaire that includes several exploratory variables assessed in the baseline questionnaire.

### Study Outcomes

#### Primary Outcome: Physical Activity

The primary outcome will be weekly MVPA minutes assessed via hip-worn accelerometers (ActiGraph wGT3X-BT). Participants will wear the accelerometers for 1-week periods throughout the 6-week intervention. Participants will be asked to wear the device every day during waking hours, removing it only for sleep or when engaging in activities involving water (eg, showering, swimming). Data from accelerometers are well-validated [64,65]. We will use self-reported MVPA minutes using the 7-day PAR as a secondary measure of physical activity.

#### Mechanisms

##### Affective Evaluations

We will use measures of affective response to exercise and intrinsic motivation for exercise to assess affective evaluations. The Feeling Scale (FS) [66] measures affective response to exercise and will be assessed during and postexercise for the in-person walking sessions each week. The FS is a single-item measure of core affect, in which participants rate their current feelings on an 11-point scale ranging from −5 (very bad) to 5 (very good). The intrinsic subscale of the Behavioral Regulations in Exercise Questionnaire (BREQ-2) [67] will be administered after listening to the audio recording in person. Intrinsic motivation is assessed with 4 items (eg, “I exercise because it’s fun”) on a 5-point response scale ranging from 0 (not true for me) to 4 (very true for me).

##### Temporal Discounting

We will use a computerized delayed discounting task [68] to assess preference for immediate versus future rewards. This task will involve participants indicating their preference for smaller quantities of money that are available immediately versus larger amounts of money available sometime in the future.

##### Action Planning

A 4-item measure of action planning [30] will be administered after participants listen to the audio recording in person each week. Items (eg, “I have made a detailed plan regarding when to exercise”) are rated on a 4-point response scale ranging from 0 (completely disagree) to 3 (completely agree).

**Figure 2:**
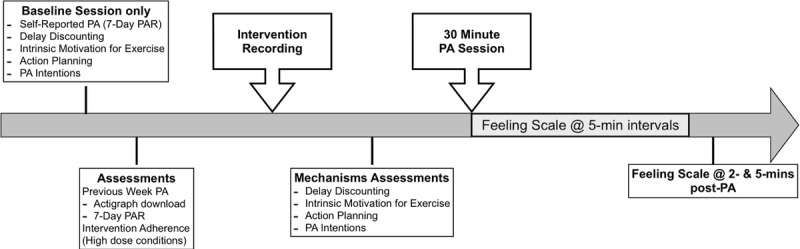
Timing of assessments, intervention delivery, and physical activity within each in-person session. PA: physical activity; PAR: Physical Activity Recall.

Figure 2 shows the assessment schedule of the key study variables.

### Intervention Adherence

All participants will listen to their assigned intervention recording during the in-person visits. For participants in the high dose condition, we will assess adherence via 2 sources: self-reports and tracking data that indicates access and time spent on the audio recording. For self-reported adherence, high dose participants will be asked at the beginning of each in-person visit to indicate how many days during the past week they listened to the audio recording.

### Optimization Decisions

Following recommendations for optimization [15], we will start by examining main effects of the 4 intervention components, provisionally include components that have a significant effect on physical activity, and provisionally exclude components that do not. Next, we will examine interactions, starting with the 2-way interactions, to determine whether any decisions about provisional inclusion and exclusion should change. We will then use participant burden and acceptability to evaluate remaining potential combinations. A combination of components that results in less time for participants will be considered preferable to a longer intervention that produces the same effect on physical activity. Only guided thinking components rated as acceptable will be included in the final intervention package.

### Statistical Analyses

#### Preliminary Analyses and Missing Data

Univariate and multivariate outliers will be identified and corrected following the data screening guidelines of Tabachnick and Fidell [69]. Patterns of missing data will be examined [70]. Multilevel modeling (MLM), which uses maximum likelihood procedures to handle missing data, will be used to address the research questions [71].

#### Analysis Plan

The first aim is to identify the optimal combination of components for meeting weekly physical activity minute guidelines. MLMs will be used to account for the nested structure of the data (ie, 6 weekly assessments nested within participants). Weekly physical activity minutes will be the dependent variable; time (weeks 0-5) will be a within-person predictor, and the main effects for each intervention component, along with all 2-way and higher order interactions, will be included as between-person predictors.

The second aim is to determine the mechanisms of each intervention component. A series of MLMs, similar to models of the first aim, will test the extent to which (A) each intervention component is associated with within-person change in its putative mechanism, and (B) the within-person change in each mechanism is prospectively associated with physical activity minutes.

### Sample Size and Power

Based on a small-to-moderate effect size (Cohen *d*=0.40) [72] and assuming a correlation of at least *r*=.40 between pre- and postintervention physical activity levels, a sample size of 176 will be sufficient to detect significant effects with 80% power and a Type I error rate of .05. This sample size estimate was generated using the Factorial Power Plan macro in SAS software, which is specifically designed to calculate power for factorial designs [73]. The sample size (N=176) will provide a sufficient number of participants while also maintaining balanced cell sizes (n) across the 16 experimental conditions, an important design feature for maintaining power for tests of the main effects and interactions [15,74]. We will enroll a total of 192 participants to account for attrition (~10%) and to maintain balanced numbers across the conditions. The factorial design is sufficiently powered for all tests, including the interactions, because the tests involve comparisons of means computed across aggregates of experimental conditions [15,73].

## Results

This project was funded by the National Cancer Institute (R21CA260360) on May 13, 2021, with a start date of May 15, 2021, and an end date of April 30, 2023. Recruitment and data collection began in February 2022, and 41 participants have enrolled in the study as of July 2022. Data collection is expected to be completed in summer 2023. Data analysis will begin after the completion of data collection.

## Discussion

### Study Implications

Delivering interventions to promote physical activity via technology-based platforms (eg, smartphone apps, websites, audio recordings) is a promising avenue to address scalability and dissemination barriers among existing interventions. The GeT Active optimization study is innovative because it identifies optimal combinations of intervention components and unpacks the “black box” in early-phase development (vs later on in the process), consistent with current frameworks of intervention development [15,20,21].

The results expected from the GeT Active optimization study will inform future refinements, testing, and use of these guided thinking tasks to promote physical activity. For example, if multiple combinations are equally effective for physical activity, this could result in a package of components that is flexible and customizable for future use (ie, individuals choose which components they want to use). Evidence on the effect of frequency of use (ie, dose) will be important in refining the intervention and subsequent dissemination, particularly if 1 component benefits from multiple uses but the others do not. We also anticipate a diverse sample with different baseline physical activity levels that will allow us to explore individual differences in the effect of the intervention components (eg, by race/ethnicity and physical activity levels).

The results from the GeT Active optimization study will also accelerate the development of the intervention more efficiently than standard approaches, which tend to either test multiple intervention components as an entire package or disparately in stand-alone studies [15]. By testing combinations of the guided thinking tasks and their frequency of use in a factorial study, we will know how the components work synergistically (or antagonistically) with each other rather than just their independent effects, as previous studies have done [17,23,26,35,41]. Moreover, by testing putative mechanisms of the components, we will be positioned to understand why components are (or are not) effective, which will help us identify which components need refinement, further optimization, or reconsideration [59].

### Limitations

The study has a few limitations. First, the intervention lasts for 6 weeks, which is a short period of time to observe effects on physical activity. We sought to balance the need to enroll a sufficient number of participants to provide a rigorous test of the guided thinking components and their combinations with a follow-up period long enough to observe sufficient variability in physical activity adherence. We are confident that 6 weeks will be long enough to provide meaningful tests of the components on physical activity, as prior studies have observed that participants begin to show meaningful variability in physical activity adherence within 4 to 6 weeks [75-77]. A longer intervention period is better suited for an efficacy trial in the final phase of intervention optimization (ie, evaluation) [15]. Second, participants in the high dose conditions receive reminders to listen to their audio recording 4 times per week via text message. These reminders could serve as an additional intervention component. Therefore, we opted to contact all participants 4 times per week via text message to increase study engagement for all participants and to ensure any potential effect of the reminders is constant across conditions. Third, the intervention dose amounts are at 2 extremes, once a week and 5 times per week. This will limit our ability to determine optimal intervention dosage. We will be able to clearly determine whether listening to the audio recording once a week is as effective as multiple times per week. If results indicate that listening multiple times per week is more effective, this will signal the importance of further specifying optimal doses in follow-up investigations.

### Conclusion

The GeT Active study will result in a scalable, audio-recorded intervention to promote physical activity that will be ready for the next stage of optimization focused on refinement and evaluation [15]. The results will accelerate progress toward the full development of a guided thinking intervention to promote physical activity.

## Appendix

Multimedia Appendix 1 National Institutes of Health (NIH) peer reviews.

## Abbreviations

BREQ-2: Behavioral Regulations in Exercise Questionnaire-2
EFT: episodic future thinking
FS: Feeling Scale
GeT: guided thinking
IPAQ: International Physical Activity Questionnaire
MLM: multilevel modeling
MOST: Multiphase Optimization Strategy
MVPA: moderate-to-vigorous physical activity
ORBIT: Obesity-Related Behavioral Intervention Trials
PAI: positive affective imagery
PAR: Physical Activity Recall
RA: research assistant

## Data Availability

The data generated during this study will be available from the corresponding author upon reasonable request.
