# Supplementary material for: Testing and Optimizing Guided Thinking Tasks to Promote Physical Activity: Protocol for a Randomized Factorial Trial
Source: JMIR Res Protoc. 2022 Sep 8;11(9):e40908. doi: 10.2196/40908 (PMC9501674; doi:10.2196/40908)
Supplement: Multimedia Appendix 1 [file resprot_v11i9e40908_app1.pdf]

SUMMARY STATEMENT
(Privileged Communication)

PROGRAM CONTACT:
Kara Hall
(240) 276-6831
hallka@mail.nih.gov

Release Date: **11/14/2020**
Revised Date:

Application Number: **1 R21 CA260360-01**

Principal Investigator

BALDWIN, AUSTIN S

Applicant Organization: SOUTHERN METHODIST UNIVERSITY

Review Group: **SPIP**
Social Psychology, Personality and Interpersonal Processes Study Section

Meeting Date: **10/15/2020**
Council: **JAN 2021**
Requested Start: **04/01/2021**
RFA/PA: **PAR19-309**
PCC: **E9HP**

Project Title: **Positive Affect, Future Thinking, and Planning: Testing Brief Intervention Components to Optimize a Novel Intervention to Promote Physical Activity**
SRG Action: **Impact Score:20 Percentile:6 +**
Next Steps: **Visit https://grants.nih.gov/grants/next_steps.htm**
Human Subjects: 30-Human subjects involved - Certified, no SRG concerns
Animal Subjects: 10-No live vertebrate animals involved for competing appl.
Gender: 1A-Both genders, scientifically acceptable
Minority: 1A-Minorities and non-minorities, scientifically acceptable
Age: 7A-Only Adults, scientifically acceptable

Project Year	Direct Costs Requested	Estimated Total Cost
1	150,000	235,230
2	125,000	196,025
TOTAL	275,000	431,255

ADMINISTRATIVE BUDGET NOTE: The budget shown is the requested budget and has not been adjusted to reflect any recommendations made by reviewers. If an award is planned, the costs will be calculated by Institute grants management staff based on the recommendations outlined below in the COMMITTEE BUDGET RECOMMENDATIONS section.

1R21CA260360-01 Baldwin, Austin

RESUME AND SUMMARY OF DISCUSSION: This exploratory grant application proposes to use a clinical trial to test the individual and combined effectiveness of technology-based approaches using positive affective imagery, episodic future thinking, and action planning for increasing levels of regular physical activity. The panel considered the application to be highly significant, based on the high proportion of individuals who continue to engage in insufficient physical activity, the adverse health consequences linked to low levels of physical activity and the limited number of effective and scalable interventions for increasing physical activity. The limited evidence supporting the use of episodic future thinking within physical activity interventions was noted as a concern. The application has numerous strengths, including its complementary and experienced investigative team with relevant expertise in all the necessary aspects of the study and its rigorous study design using the Multiphase Optimization Strategy (MOST) framework to efficiently test combinations of the three approaches. Reviewers considered the analytic approach using hierarchical linear models to be a strength of the design, although there were some concerns with the adequacy of the statistical power to explore the expected interactions. Following the discussion, the panel agreed that the applications' high significance and numerous strengths outweighed any weaknesses, resulting in a study with a high potential impact on the field.

DESCRIPTION (provided by applicant): Insufficient levels of regular physical activity (< 150 minutes/week of moderate-to-vigorous physical activity [MVPA]) are associated with various health risks, including increased risk of breast, colon, and endometrial cancers. Nearly 50% of adults in the US report insufficient levels of regular physical activity (PA), and 26.6% report no regular PA at all. Most current PA interventions are resource intensive (i.e., cost to implement, participant time and burden) or "black boxes" where the active intervention components are unclear, creating critical barriers to scalability and dissemination and limiting their impact on population health and cancer prevention. Novel intervention strategies to increase regular PA need to be both effective and scalable. Delivering PA interventions via technology-based platforms can address scalability and dissemination barriers by minimizing cost and resource demands; however, current technology-based PA interventions also have the same "black box" problem as other interventions. Three brief intervention techniques are promising candidate components to consider in developing and optimizing a novel PA intervention to address existing barriers to scalability and dissemination: positive affective imagery (PAI), episodic future thinking (EFT), and action planning (AP). Optimal component dosage is also important for optimizing an intervention. Guided by the Multiphase Optimization Strategy (MOST) framework, we will develop audio-recorded PAI, EFT, and AP components and enroll insufficiently active (i.e., < 150 minutes/week of MVPA) adults (N=192) in a 6-week factorial intervention to test different component combinations, different doses, and their putative mechanisms to optimize a novel, scalable intervention to promote PA. In Aim 1, we will identify the optimal combination of components for meeting weekly PA minute guidelines by testing the independent and interactive effects of the components on weekly PA minutes during the 6-week intervention. In Aim 2, we will determine the mechanisms of each intervention component by testing the extent to which (a) each component changes its putative mechanism, and (b) within-person changes in each mechanism are associated with weekly PA minutes. The research will result in an optimized, audio-recorded PA intervention that is effective and scalable and will position our interdisciplinary team to conduct a future randomized controlled trial (RCT) focused on refinement and evaluation. These outcomes will accelerate progress toward developing an intervention to promote PA that has potential for immediate and broad impact on population health and cancer prevention.

PUBLIC HEALTH RELEVANCE: Insufficient levels of regular physical activity are associated with various health risks, including increased risk of breast, colon, and endometrial cancers. Current rates of regular physical activity among US adults are suboptimal and novel interventions to promote physical

activity are needed to have a broad impact on population health and cancer prevention. This study will result in an effective and scalable audio-recorded intervention that is optimized to promote physical activity and ready for further refinement and evaluation.

CRITIQUE 1

Significance: 1
Investigator(s): 1
Innovation: 2
Approach: 2
Environment: 1

Overall Impact: This is a new R21 proposal to NCI from a group led by investigators at Southern Methodist University (SMU) building a scalable physical activity intervention from three theory-driven components within a Multiphase Optimization Strategy (MOST) trial. Aim 1 is to identify the optimal combination of components of an effect online physical activity intervention over 6 weeks, and Aim 2 is to identify the mechanisms of each component. A total of 192 participants will be randomized to one of 16 conditions defined by the 2x2x2x2 factorial design crossing three intervention components (affective imagery, episodic future thinking, action planning) and two dosage levels (low/high) and physical activity and hypothesized mechanisms will be assessed once per week. The significance of identifying low-cost, scalable, and theoretically grounded intervention components that are hypothesized to operate via distinct mechanisms and therefore might be synergistic is quite high. Many people could benefit from the results of this project should it be successful given the very high prevalence of insufficient PA. The approach is rigorous and aligns tightly with the theoretical framework, with only minor noted limitations. The clarity and precision of the theoretical model is highly innovative, as are many aspects of the design including the MOST framework and the assessment of component mechanisms. The team is very strong and has collaborated before and the environments are sound. Overall, this is deemed to be a high impact proposal.

1. Significance:

Strengths

- Insufficient physical activity (PA) is a major public health burden and is highly prevalent across demographic groups in the US.
- Existing interventions are often difficult to scale, so developing intervention components that are eventually scalable is highly significant.
- The focus on mechanisms increases the significance of the work because it enables future programs to be developed (e.g., targeting specific populations) that engage the same mechanisms even if they are not identical at the surface level.
- Examining dosage is also significant because it enables the eventual development more cost-effective interventions.
- The fact that each of the three components targets different psychological mechanisms of action, and at different phases of the behavior change process, provides strong rationale to look at each of them individually and in interaction.

Weaknesses

- Despite the strength that low PA is pervasive across the population, there are still racial and ethnic disparities in PA. There is little consideration of these in the proposal.

2. Investigator(s):

Strengths

- The PI, Baldwin, is Associate Professor of Psychology at SMU and a member of the Simmons Cancer Center at the University of Texas Southwestern MC. He has strong experience in brief, tech-based physical activity interventions. He also has expertise in the theoretical models and mechanisms under examination in the proposed work.
- Co-I Martin is Associate Professor of Population Health at the University of Kansas Medical Center (KUMC) and Associate Director of the imaging center there. Martin brings expertise in the roles of self-regulation and reward processing in health-related decisions.
- Co-I Levens is Associate Professor of Psychological Science at University of North Carolina at Charlotte (UNCC) has expertise in emotion, emotion regulation, and executive function as well as behavioral task design relevant to the proposed work.
- Co-I Kouros is Associate Professor of Psychology at SMU and will serve as the biostatistician on the project. Kouros has extensive expertise conducting and publishing advanced statistical methods including those to be deployed in the proposed work.
- The PIs have collaborated effectively in the past on projects, manuscripts, and grants.
- The team has experience managing federal funds and large-scale projects.

Weaknesses

- None noted.

3. Innovation:

Strengths

- The focus on scalability from the very beginning of intervention development is innovative.
- The clear, theory-driven, mechanistic orientation of the research is innovative.
- The use of the MOST framework to test multiple components simultaneously is innovative and increases efficiency.
- The ability of MOST to also identify ineffective components is also highly innovative.

Weaknesses

- None noted.

4. Approach:

Strengths

- The MOST framework is a strength of the approach because it increases efficiency and affords a test of many potential combinations of the three components and dosages.
- The preliminary data are strong and provide evidence not only of the rigor of the work that forms the basis of the scientific premise but also of the feasibility of several of the components and their (individual) efficacy for influencing PA and of the team collaborating.

- The power analysis is rigorous and the sample size (N = 192) is well justified.
- The assessment of PA using accelerometer and recall is rigorous.
- The study protocol is detailed and generally well controlled.
- The assessment of the hypothesized intervention mechanisms is specific and rigorous.
- The analytic strategy and especially the carefully detailed chain of optimization decisions is a strength of the design.
- The use of audio recordings is well justified and a strength of the design because it increases feasibility, scalability, and accessibility, and reduces complexity.

Weaknesses

- Episodic future thinking (EFT) has not yet been shown to increase PA, but this limitation is offset by its efficacy for changing other health behaviors and the strong theoretical rationale for its hypothesized effects on PA.
- A limitation of the high/low dosage manipulation is that participants in the low dose condition only hear the intervention in the lab, whereas those in the high dose condition can listen to the intervention on their phone from anywhere. So, there is a slight confound between dosage and location/familiarity/accessibility.
- It is not clear what the implication of the in-lab PA session is for generalizability. It is possible that being offered the opportunity to exercise on a weekly basis could “jump start” physical activity for participants in some of the conditions, but that effect might not hold for people not afforded the opportunity to do so. (Though it is important to note that this feature of the design seems necessary to measure process-level variables such as affective evaluations of PA.)

5. Environment:

Strengths

- The environment at SMU is strong and can support the proposed work.
- The environment at the sites of the Co-Is, including University of North Carolina at Charlotte and University of Kansas Medical Center (KUMC).

Weaknesses

- None noted.

Study Timeline:

Strengths

- Long recruitment period is a strength.
- Overall the timeline is feasible.

Weaknesses

- Only 3 months for ramp-up before recruitment begins, including building and refining the intervention materials, might be unrealistic.

Protections for Human Subjects:

Acceptable Risks and/or Adequate Protections

- All potential risks addressed, including physical injury or other adverse effects.

Data and Safety Monitoring Plan (Applicable for Clinical Trials Only):

Acceptable

- Thoughtful and appropriate.

Inclusion Plans:

- Sex/Gender: Distribution justified scientifically
- Race/Ethnicity:
- For NIH-Defined Phase III trials, Plans for valid design and analysis: Not applicable
- Inclusion/Exclusion Based on Age: Distribution justified scientifically
- Inclusion of all genders scientifically justified.
- Inclusion of diverse groups following a distribution similar to the Dallas County census data is justified. There is a reasonable plan for enhancing participation by ethnic minority individuals.
- Exclusion of adults over the age of 64 is justified by the potential risks of moderate-to-vigorous PA for older adults and the attendant need for enhanced safety protocols.

Vertebrate Animals:

Not Applicable (No Vertebrate Animals)

Biohazards:

Not Applicable (No Biohazards)

Resource Sharing Plans:

Not Applicable (No Relevant Resources)

Budget and Period of Support:

Recommend as Requested

CRITIQUE 2

Significance: 3
Investigator(s): 2
Innovation: 2
Approach: 3
Environment: 1

Overall Impact: This application proposes a clinical trial to increase physical activity among adults who are not currently active. The trial will manipulate three components that have been shown previously to change behavior (Positive Affective Imagery, Episodic Future Thinking, Action Planning) and cross it with dosage of the intervention (1 time/week versus 5 times/week). It will also examine the hypothesized mechanism of change for each of the manipulated components. The project is significant and innovative in that it seeks to identify components of the intervention and the mechanism of action

for each component. If successful, it will provide needed information on why a specific component increases engagement in physical activity. The intervention is also relatively brief and delivered via audio recordings on a smart phone, so if successful the intervention has the potential to be scalable. The investigative team is excellent with a history of successful collaboration. The environment is sufficient for this research. Limitations include lack of consideration of moderators of intervention effectiveness, power to test mediators, and the daily contacts may be an additional component of the intervention that explains its effectiveness that would not be scalable. These limitations are minor compared with the potential of what can be learned from this research.

1. Significance:

Strengths

- An effective and scalable intervention for physical activity would be highly significant.
- Manipulates three components that have shown individually shown to be relevant for health: Positive Affective Imagery, Episodic Future Thinking, Action Planning. Positive Affective Imagery and Action Planning have further been shown previously to be effective at changing physical activity.
- Each component is linked to a specific mechanism thought to be responsible for the behavior change. Identifying the mechanism of change as well as target is highly significant.

Weaknesses

- There is no consideration of individual differences that might moderate intervention effectiveness (e.g., differences across age, gender, race, personality, etc.)
- Episodic future thinking is a novel target that has been a target in interventions to change behavior but its efficacy for behavior change, particularly in the context of an intervention for physical activity, is not yet known.

2. Investigator(s):

Strengths

- PI Baldwin in an expert in mechanisms of behavior change and has experience running clinical trials.
- Co-I Martin has expertise in temporal discounting and applying it to behavior change.
- Co-I Levens has expertise in emotion regulation.
- Co-I Kouros will serve as the data analyst and has previous experience in this role on other funded projects.
- The investigative team previously worked together on a funded project to test a guided imagery intervention to increase healthy eating behaviors. The team thus has a history or working together.

Weaknesses

- Not clear that co-I Kouros has experience analyzing data from trials.

3. Innovation:

Strengths

- Addresses how the three components potentially interact with each other, which may either amplify or dampen effects of the individual components.
- Crosses each of the components with dosage to identify the effective amount for each component.

Weaknesses

- The targets of intervention are not innovative nor is the delivery method.

4. Approach:

Strengths

- The use of 16 possible combinations to isolate which components and doses are effective.
- The use of accelerometers to measure an objective physical activity outcome.
- Addresses the number of doses needed for the intervention to be effective.
- Tests three components of an intervention and the hypothesized mediators that would explain why the intervention works.
- The intervention is delivered through brief audio recordings through a smart phone.

Weaknesses

- Study is powered to detect effects of the intervention on physical activity but not the mediation analysis.
- The dosage amount is at two extremes. Such information is helpful for knowing whether there needs to be more than a minimal dosage or whether a high dosage is effective but does not say anything about optimal dosage.
- In the Approach, the investigators cite their intervention on positive affective imagery and episodic future thinking to change food choices as evidence for these factors within a health context. The cited paper reports the protocol and no findings. Thus, although it shows that the investigators can develop an intervention, it does not provide evidence that it can change behavior.
- The daily contacts during the intervention, although the same for all participants, may also be a component of the intervention that contributes to efficacy.

5. Environment:

Strengths

- Southern Methodist University has the necessary resources for this research.

Weaknesses

- None noted.

Study Timeline:

Strengths

- The PI has experience with running trials that have the enrollment of about 12 participants/month. If this experience is maintained, the proposed trial is feasible within the 2-year timeframe of the award.

Weaknesses

- None noted.

Protections for Human Subjects:
Acceptable Risks and/or Adequate Protections
Data and Safety Monitoring Plan (Applicable for Clinical Trials Only):
Acceptable

Inclusion Plans:

- Sex/Gender: Distribution justified scientifically
- Race/Ethnicity: Distribution justified scientifically
- For NIH-Defined Phase III trials, Plans for valid design and analysis: Not applicable
- Inclusion/Exclusion Based on Age: Distribution justified scientifically

Vertebrate Animals:
Not Applicable (No Vertebrate Animals)

Biohazards:
Not Applicable (No Biohazards)

Resource Sharing Plans:

- Not reported.

Budget and Period of Support:
Recommend as Requested

CRITIQUE 3

Significance: 2
Investigator(s): 2
Innovation: 1
Approach: 2
Environment: 2

Overall Impact: This is a very strong and well conceptualized proposal that aims to address scalability and dissemination barriers to interventions that are likely to be useful in increasing physical activity (PA) among adults. The public health significance of the proposed work is very clear, inasmuch as low PA is associated with diverse health risks. The intervention strategies proposed are highly compelling as brief interventions that also show established influences on health behaviors. The key innovation of the proposal is to consider three specific intervention components in concert, both in terms of main effects and in terms of potential interactions. Limitations are modest and consistent with the scope of the study. Specifically, power analyses are based on optimistic effect sizes, and the overall proposed sample size

is modest given the potential complexity of interactive effects. Nevertheless, construed as a two-year project that can generate preliminary data for further research, the project is highly compelling and likely to yield valuable insights into strategies for increasing PA.

Study Timeline:

Strengths

- The timeline is realistic for the proposed research.

Weaknesses

- None noted.

Protections for Human Subjects:

Acceptable Risks and/or Adequate Protections

- No concerns.

Data and Safety Monitoring Plan (Applicable for Clinical Trials Only):

Acceptable

- No concerns.

Inclusion Plans:

- Sex/Gender: Distribution justified scientifically
- Race/Ethnicity: Distribution justified scientifically
- For NIH-Defined Phase III trials, Plans for valid design and analysis: Not applicable
- Inclusion/Exclusion Based on Age: Distribution justified scientifically

Vertebrate Animals:

Not Applicable (No Vertebrate Animals)

Biohazards:

Not Applicable (No Biohazards)

Resource Sharing Plans:

Not Applicable (No Relevant Resources)

Budget and Period of Support:

Recommend as Requested

THE FOLLOWING SECTIONS WERE PREPARED BY THE SCIENTIFIC REVIEW OFFICER TO SUMMARIZE THE OUTCOME OF DISCUSSIONS OF THE REVIEW COMMITTEE, OR REVIEWERS' WRITTEN CRITIQUES, ON THE FOLLOWING ISSUES:

PROTECTION OF HUMAN SUBJECTS: ACCEPTABLE

INCLUSION OF WOMEN PLAN: ACCEPTABLE

INCLUSION OF MINORITIES PLAN: ACCEPTABLE

INCLUSION ACROSS THE LIFESPAN: ACCEPTABLE

COMMITTEE BUDGET RECOMMENDATIONS: The budget was recommended as requested.

Footnotes for 1 R21 CA260360-01; PI Name: Baldwin, Austin S

+ Derived from the range of percentile values calculated for the study section that reviewed this application.

NIH has modified its policy regarding the receipt of resubmissions (amended applications).See Guide Notice NOT-OD-18-197 at https://grants.nih.gov/grants/guide/notice-files/NOT-OD-18-197.html. The impact/priority score is calculated after discussion of an application by averaging the overall scores (1-9) given by all voting reviewers on the committee and multiplying by 10. The criterion scores are submitted prior to the meeting by the individual reviewers assigned to an application, and are not discussed specifically at the review meeting or calculated into the overall impact score. Some applications also receive a percentile ranking. For details on the review process, see http://grants.nih.gov/grants/peer_review_process.htm#scoring.

MEETING ROSTER

Social Psychology, Personality and Interpersonal Processes Study Section
Risk, Prevention and Health Behavior Integrated Review Group
CENTER FOR SCIENTIFIC REVIEW
SPIP
10/15/2020 - 10/16/2020

Notice of NIH Policy to All Applicants: Meeting rosters are provided for information purposes only. Applicant investigators and institutional officials must not communicate directly with study section members about an application before or after the review. Failure to observe this policy will create a serious breach of integrity in the peer review process, and may lead to actions outlined in NOT-OD-14-073 at https://grants.nih.gov/grants/guide/notice-files/NOT-OD-14-073.html and NOT-OD-15-106 at https://grants.nih.gov/grants/guide/notice-files/NOT-OD-15-106.html, including removal of the application from immediate review.

CHAIRPERSON(S)

KRUEGER, ROBERT F, PHD
DISTINGUISHED MCKNIGHT UNIVERSITY PROFESSOR
DEPARTMENT OF PSYCHOLOGY
UNIVERSITY OF MINNESOTA
MINNEAPOLIS, MN 55455

MEMBERS

BERG, CYNTHIA A, PHD
DISTINGUSHED PROFESSOR AND DEAN
DEPARTMENT OF PSYCHOLOGY
UNIVERSITY OF UTAH
SALT LAKE CITY, UT 84112

BERKMAN, ELLIOT TODD, PHD
PROFESSOR
DEPARTMENT OF PSYCHOLOGY
UNIVERSITY OF OREGON
EUGENE, OR 97403

BIRDITT, KIRA S, PHD
RESEARCH ASSOCIATE PROFESSOR
THE INSTITUTE FOR SOCIAL RESEARCH
UNIVERSITY OF MICHIGAN
ANN ARBOR, MI 48106

EKSTRAND, MARIA L, PHD
PROFESSOR
DIVISION OF PREVENTION SCIENCE
DEPARTMENT OF MEDICINE
UNIVERSITY OF CALIFORNIA SAN FRANCISCO
SAN FRANCISCO, CA 94143

ERICKSON, KIRK I, PHD
PROFESSOR
DEPARTMENT OF PSYCHOLOGY
UNIVERSITY OF PITTSBURGH
PITTSBURGH, PA 15260

FAGUNDES, CHRISTOPHER PAUL, PHD
ASSOCIATE PROFESSOR
DEPARTMENT OF PSYCHOLOGICAL SCIENCES
RICE UNIVERSITY
HOUSTON, TX 77005

GAGE-BOUCHARD, ELIZABETH, PHD
ASSOCIATE PROFESSOR
DEPARTMENT OF CANCER PREVENTION AND CONTROL
ROSWELL PARK COMPREHENSIVE CANCER CENTER
BUFFALO, NY 14263

GRAHAM-ENGELAND, JENNIFER ELISE, PHD
ASSOCIATE PROFESSOR
DEPARTMENT OF BIOBEHAVIORAL HEALTH
THE PENNSYLVANIA STATE UNIVERSITY
UNIVERSITY PARK, PA 16802

GUTCHESS, ANGELA, PHD *
ASSOCIATE PROFESSOR
DEPARTMENTS OF NEUROSCIENCE AND PSYCHOLOGY
BRANDIES UNIVERSITY
WALTHAM, MA 02453

HALL, WILLIAM JAMES, PHD *
ASSISTANT PROFESSOR
SCHOOL OF SOCIAL WORK
UNIVERSITY OF NORTH CAROLINA CHAPEL HILL
CHAPEL HILL, NC 27599-3550

INFURNA, FRANK JOHN, PHD *
ASSOCIATE PROFESSOR
DEPARTMENT OF PSYCHOLOGY
ARIZONA STATE UNIVERSITY
TEMPE, AZ 85281

KIM, YOUNGMEE, PHD
PROFESSOR
DEPARTMENT OF PSYCHOLOGY
UNIVERSITY OF MIAMI
CORAL GABLES, FL 33146

LANGER, SHELBY, PHD
ASSOCIATE PROFESSOR
EDSON COLLEGE OF NURSING AND HEALTH INNOVATION
ARIZONA STATE UNIVERSITY
PHOENIX, AZ 85004

LEWIS, TENÉ T, PHD
ASSOCIATE PROFESSOR
DEPARTMENT OF EPIDEMIOLOGY
ROLLINS SCHOOL OF PUBLIC HEALTH
EMORY UNIVERSITY
ATLANTA, GA 30322

LIN, FENG VANKEE, PHD
ASSOCIATE PROFESSOR
SCHOOL OF MEDICINE AND DENTISTRY
UNIVERSITY OF ROCHESTER
ROCHESTER, NY 14642

MANNING, MARK A, PHD *
ASSISTANT PROFESSOR
DEPART OF ONCOLOGY
WAYNE STATE UNIVERSITY
DETROIT, MI 48201

MARGRETT, JENNIFER ANN, PHD *
PROFESSOR
DIRECTOR OF THE GERONTOLOGY PROGRAM
DEPARTMENT OF HUMAN DEVELOPMENT AND
FAMILY STUDIES
IOWA STATE UNIVERSITY
AMES, IA 50011

MUILENBURG, JESSICA L., PHD *
ASSOCIATE PROFESSOR AND GRADUATE COORDINATOR
DEPARTMENT OF HEALTH PROMOTION AND BEHAVIOR
COLLEGE OF PUBLIC HEALTH
UNIVERSITY OF GEORGIA
ATHENS, GA 30602

PACHANKIS, JOHN EDWARD, PHD
ASSOCIATE PROFESSOR
DEPARTMENT OF SOCIAL AND BEHAVIORAL SCIENCES
YALE SCHOOL OF PUBLIC HEALTH
YALE UNIVERSITY
NEW HAVEN, CT 06520

SALAZAR, LAURA F, PHD
PROFESSOR & DIRECTOR OF PHD PROGRAM
SCHOOL OF PUBLIC HEALTH
DEPARTMENT OF HEALTH POLICY & BEHAVIORAL SCIENCE
GEORGIA STATE UNIVERSITY
ATLANTA, GA 30302

SCHERER, LAURA D., PHD, MA *
ASSISTANT PROFESSOR
UNIVERSITY OF COLORADO SCHOOL OF MEDICINE
AURORA, CO 80045

SUTIN, ANGELINA R, PHD
PROFESSOR
DEPARTMENT OF BEHAVIORAL SCIENCES AND
SOCIAL MEDICINE
COLLEGE OF MEDICINE
FLORIDA STATE UNIVERSITY
TALLAHASSEE, FL 32306

WILLIAMS, MICHELLE K, PHD
ASSOCIATE VICE PRESIDENT AND ASSOCIATE PROFESSOR
DEPARTMENT OF PSYCHOLOGICAL SCIENCES
UNIVERSITY OF CONNECTICUT
STORRS, CT 06269

WILSON, DAWN K, PHD
PROFESSOR
DEPARTMENT OF PSYCHOLOGY
UNIVERSITY OF SOUTH CAROLINA
COLUMBIA, SC 29208

SCIENTIFIC REVIEW OFFICER

BOULAY, MARC, PHD
SCIENTIFIC REVIEW OFFICER
CENTER FOR SCIENTIFIC REVIEW
NATIONAL INSTITUTES OF HEALTH
BETHESDA, MD 20892

EXTRAMURAL SUPPORT ASSISTANT

NJUKI, JENNIFER N, BS
EXTRAMURAL SUPPORT ASSISTANT
CENTER FOR SCIENTIFIC REVIEW
NATIONAL INSTITUTES OF HEALTH
BETHESDA, MD 20892

* Temporary Member. For grant applications, temporary members may participate in the entire meeting or may review only selected applications as needed.

Consultants are required to absent themselves from the room during the review of any application if their presence would constitute or appear to constitute a conflict of interest.
